# Experimental Investigation on Mechanical Properties of Geocell Strips at Low Temperature

**DOI:** 10.3390/ma15155456

**Published:** 2022-08-08

**Authors:** Qiyu Bai, Guofeng He, Yong Wang, Jie Liu

**Affiliations:** 1 College of Water and Architectural Engineering, Shihezi University, Shihezi 832003, China; 2 Xinjiang Transportation Planning Surveying and Design Institute Co., Ltd., Urumqi 830006, China

**Keywords:** road engineering, geocell strip, low temperature, tensile strength

## Abstract

Geocell is widely used in the treatment of poor roadbed, which can restrain soil laterally and improve the stability of soil. In cold area engineering, a change in temperature can influence the mechanical properties of geocell of different materials. To study the mechanical response of geocell at low temperatures, three types of geocell strips commonly used in engineering, namely the polyethylene (HDPE), polypropylene (PP), and polyester (PET), were studied via the uniaxial tensile test at the ambient temperatures of −5 °C, −20 °C, and −35 °C, respectively. Meanwhile, the tensile strength, fracture mode, and temperature sensitivity of geocell specimens were compared. It is concluded that: (1) at low temperatures, the tensile strengths of HDPE and PET geocell strips are significantly improved, while that of the PP geocell strip is less sensitive to the temperature. (2) The PP geocell is subject to a brittle failure at all ambient temperatures. The PET geocell strip experiences a hard-ductile failure at normal temperatures of −5 °C and −20 °C. While in the tensile test at −35 °C, it is prone to brittle failure and hard-ductile failure. The HDPE geocell strip suffers from ductile failure at all ambient temperatures. (3) At low temperatures, overall, the tensile properties of the PET geocell strip is better than those of the PP and HDPE geocell strips.

## 1. Introduction

Geocell is a special three-dimensional mesh geosynthetic, which is widely used in subgrade, side slope, and retaining wall engineering [1,2,3,4,5,6]. Geocell is embedded in soil as a reinforced material. Using a cell wall, the soil stability can be effectively improved by changing the stress diffusion of surrounding soil to achieve a long-term constraint to the lateral displacement of soil particles [7,8,9]. The raw materials of geocell are mainly high-density polyethylene (HDPE), polypropylene (PP), and polyester (PET), which are made through a series of processes, such as the extrusion or stretching of polymer [10]. The basis for a performance test of geocell in China is mainly the “Test Regulation for Geosynthetics in Highway Engineering” (JTG E50-2006) [11] and the test methods in “Geosynthetics Plastic Geocell” (GB/T 19274-2003) [12] and “Geosynthetics Plastic Geogrid” (GB/T 17689-2008) [13] are used as references.

At present, research into geocell mainly focus on the reinforcement effect, action mechanism, and mechanical properties of geocell. Yang [14] concluded that the geocell can constrain the lateral deformation and stress diffusion of soil through an indoor model test on the loess foundation reinforced by geocell and presented the control parameters for engineering application and the calculation formula of foundation bearing capacity. Sun [15] analyzed the influences of geocell welding distance, soil compactness, and other factors on the stability of geocell reinforced embankment through an indoor test and concluded that the geocell reinforced embankment could effectively reduce the lateral displacement of the slope surface and improve the bearing capacity of the embankment. Through a model load test on the reinforced and unreinforced sand layer, Sujit Kumar Dash [16] considered that the greater the density of filler, the more obvious the reinforcement effect of geocell on foundation soil. Zhou [17] studied the effect of geocell reinforcement on a soft soil foundation by the indoor model test and reported that the settlement of soft soil foundation decreased significantly under the action of a geocell reinforced sand cushion. Li [18] concluded that the tensile rate had a great influence on the strain of geocell strip through a tensile test on the HDPE geocell strip. Masahiro Shinoda [19] used the uniaxial tensile test to analyze the influence of length–width ratio on the geogrid specimens made of different materials by changing the tensile rate, specimen length, and specimen width. It was found that the tensile rate had a great influence on the stress–strain response of polyolefin specimens, while the sensitivity of polyester material specimens to the tensile rate was small. Through a biaxial tensile test on geocell, Ma [20] concluded that compared with the strength of the strip, the strength of the joint was dominant in determining the overall strength of the geocell. When the overall elongation was constant, the larger the geocell height was, the smaller the elongation was. Yang [21] compared and analyzed the mechanical properties of the HDPE, PP, and PET through a tensile test on a geocell strip and concluded that the geocell strip with PET as the raw material had the advantages of polyolefin material. Through a biaxial tensile test on geocell, Zhang [22] analyzed the factors affecting the overall strength of geocell. The order of influence degree from large to small is the cell height, the welding distance, and the tensile speed. Wang [23] carried out uniaxial tensile tests on HDPE unidirectional geogrid splines with different strengths by using the single rib method at low temperature and studied the relationship between the tensile force and the elongation of geogrid when temperature served as a variable. It was found that the tensile strength of geogrid was significantly improved with a decrease in temperature. Amjadi [24] carried out uniaxial tensile test on high density polyethylene. The sensitivity of the material to temperature was investigated at −40 °C, 23 °C, 53 °C, and 82 °C. The experimental results show that the tensile strength of HDPE increases significantly with the decrease of temperature. Hsueh [25] studied the tensile mechanical behavior of HDPE material under UV irradiation and temperature. There is a linear relationship between elongation-at-failure and molar mass. When UA intensity is higher than 40% (61 W/m^2^), the ductility of the samples is completely lost, and embrittlement occurs. Bilim [26] tested the low temperature performance of polypropylene composite strip. Tensile tests were carried out at −196 °C. The results show that the PP complex has good ductility at low temperature and is suitable for low temperature environment.

The engineering application in cold regions shows that the geocell as a flexible reinforced structure [27,28] has strong advantages in inhibiting the thawing settlement and the frost heave of subgrade [29,30,31,32]. Due to the extreme cold in winter in the Xinjiang region of China, according to our monitoring data of a typical road surface in the Xinjiang region. the highest temperature in winter remains at about −5 °C, and the lowest temperature can reach about −30 °C. Therefore, we tested the low temperature performance of materials according to the measured temperature of engineering. Those temperatures can better reflect the service performance of geocell in engineering practice. In cold regions, the geocell is in a low temperature state for long periods. As an organic polymer material, geocell is different in sensitivity to temperature when its material is different [33,34]. In low temperatures, the change mechanism of material properties is not clear. This may lead to engineering design errors, affect the engineering service performance, and even cause engineering accidents. Based on the existing research results, scholars mostly study the mechanical properties of geocell strips at conventional temperatures. There are still some deficiencies in the research into the mechanical properties of geocell strips under a coupling effect of loading and low temperature. This fact limits the popularization and application of geocell reinforcement structure in cold regions.

In this paper, a tensile testing machine that can achieve high and low temperatures was used to study the variation law of strength and deformation of geocell strips, with the temperature and material as variables. The scanning electron microscope (SEM) was used to analyze the micro fracture of geocell strips, and the influence of low temperature on the mechanical properties of geocell strips was studied. The curve characteristics, failure mode, and temperature sensitivity of the geocell strips of three different materials were analyzed in detail. Finally, the materials with more advantages in low temperature environment were selected. The experimental results can provide a theoretical basis for the application of geocell in cold regions.

## 2. Materials and Methods

### 2.1. Materials

All the geocell strips studied in this paper were produced from Anhui Huifeng New Synthetic Material Co., Ltd., Hefei, China. In order to fit with the actual project, those strips are the most commonly used size on the market. The tensile strength of HDPE strips is generally lower than PP and PET strips. Therefore, the thickness of the HDPE strip is slightly greater than its two counterparts under the premise of ensuring its economic practicability. The HDPE, PP, and PET were used as the raw materials for preparing geocell specimens, and their specific parameters are shown in Table 1.

### 2.2. Test Method

**Figure 1:**
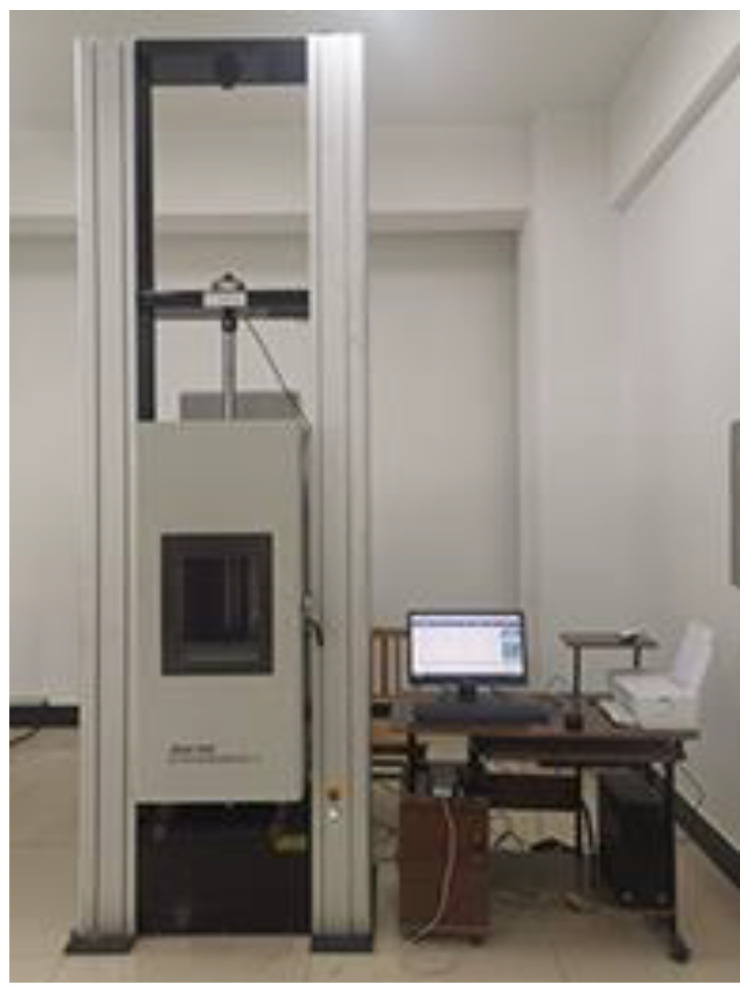
The YG028HS electronic machine with controlled temperature.

The YG028HS electronic machine was adopted in the test, as shown in Figure 1. Temperature can be controlled in the test machine, and its maximum tensile force is 50 kN. The effective test range of the temperature box is 0–800 mm, the temperature control range is −60–150 °C, and the resolution of the temperature control table is 0.1 °C.

**Figure 2:**
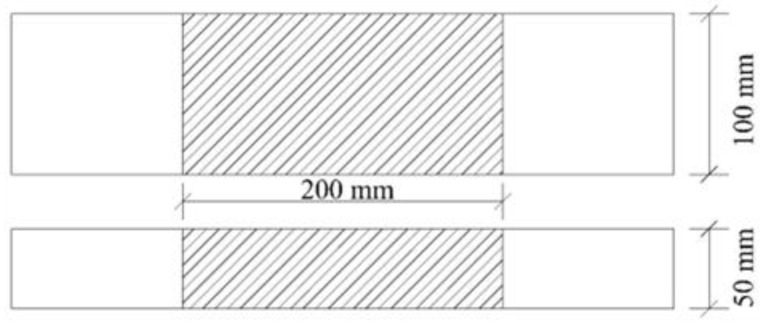
The specimen sizes of type I (100 mm) and II (50 mm).

To study the influence of temperature on the mechanical properties of geocell specimens made of the three raw materials, the ambient temperatures were set to −5 °C, −20 °C, and −35 °C. and the results were compared with that of specimens under 20 °C (room temperature). The tensile rate is 20 mm/min. There are two types of heights of the specimens: 100 mm (type I) and 50 mm (type II). The tensile strength is equal to the ratio of the peak stress to the cross-sectional area at a certain point. That is, tensile strength is theoretically independent of the material size. However, to ensure the data reliability and more objectively study the influence of temperature on the material properties, this paper uses two kinds of geocell specimens for comparison. According to the “Test Code for Geosynthetics in Highway Engineering” (JTG E50-2006) [11], the gauge area (including the centerline distance of jig between the upper and lower) is 200 mm (Figure 2). To better compare the various differences in the three raw materials during tension, other factors were kept unchanged, and five groups of tests were conducted for a parallel comparison.

## 3. Results and Discussion

Before carrying out the tests under different temperatures, the stress–strain relationship of three types of geocell specimens at room temperature was analyzed. It was found that the width of specimens had little effect on the tensile strength. The stress–strain curves in the present study are consistent with those of the dumbbell-shaped specimens made of the three raw materials in existing literature [21]. This indicates that the long strip specimen was feasible. When comparing the tensile properties of the three raw materials, the HDPE and PET specimens were selected as group I and group II with lower tensile strength, and PP specimens were selected as group II with more concentrated stress–strain curves.

### 3.1. Stress–Strain Relationship and Fracture Mode of HDPE Specimens

**Figure 3:**
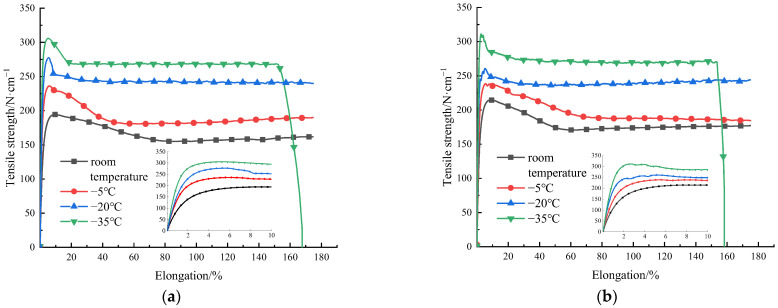
Tension curves of HDPE specimens I (**a**) and II (**b**) respectively at different temperatures. (Note: The small figure shows the detail of the first 10% elongation of the tension curves of HDPE specimens).

**Figure 4:**
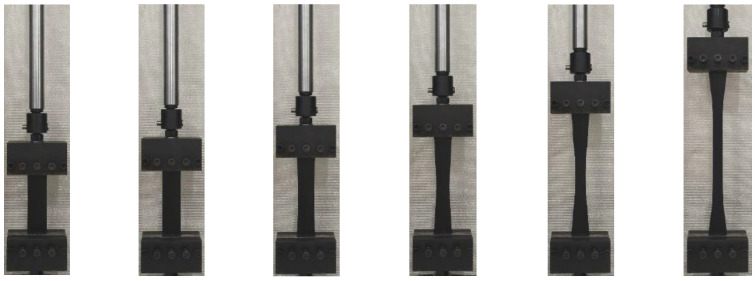
The tension process of a HDPE specimen.

The stress–strain relationships at different temperatures and the tension process of HDPE specimens are shown in Figure 3 and Figure 4, respectively. The trend of stress–strain relationships of type I and type II specimens is roughly the same. At the early stage of tension, the specimen is in an elastic stage, and the stress–strain relationship is approximately linear increasing. With the increase of elongation, the peak stress appears on the stress–strain relationship. Subsequently, strain softening occurs in the specimen, and its stress shows a downward trend. Meanwhile, a necking appears in the specimen, and the specimen becomes thinner with a narrower width. The residual parts of the specimen are pulled at positions where the orientation hardening is first reached. During this process, the stress is basically unchanged, but the elongation increases, namely, the cold tensile stage is reached. The reason is that the production process of HDPE geocell is extrusion and the material is not fully oriented. It also shows that the HDPE specimen is ductile. Due to a favorable ductility, fracture does not occur during the tension process of the specimen at the room temperature, −5 °C, and −20 °C. When the maximum displacement of the equipment reaches 350 mm, the tension is stopped. During the tension process at a temperature of −35 °C, the specimen fractures. The reason is that a low temperature reduces the molecular energy inside the material, and the flexibility of the molecular chains decreases with a decrease in temperature. Thus, the configuration of the molecular chain changes, which improves the stiffness and reduces the elongation of the material. Finally, a fracture of the chain segment causes the fracture of molecular chain, macroscopically, and thus the specimen fractures.

**Figure 5:**
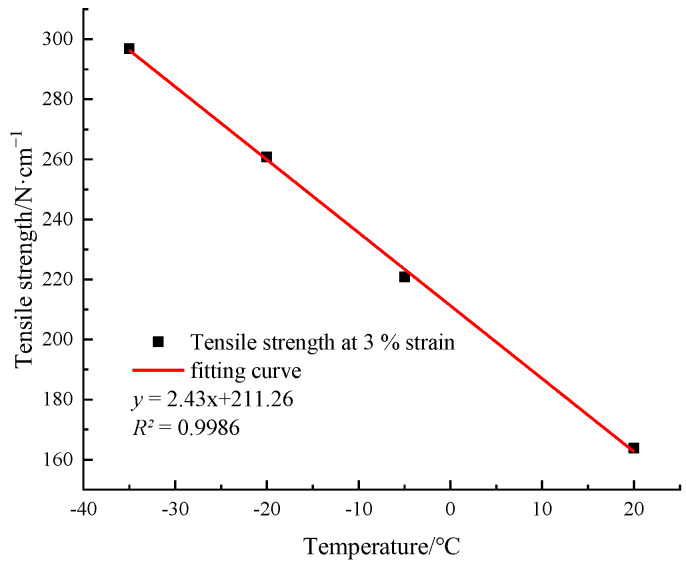
The tensile strength–temperature curve of HDPE specimen.

According to the experimental data in Figure 3, with a decrease in temperature, the overall tensile strength of the HDPE I and II specimens is significantly improved, which indicates that the HDPE material is sensitive to temperature. In addition, the ambient temperature of type I and type II specimens decreases from the room temperature to −35 °C, and their yield strengths increase by 57.5% and 45%, respectively. To objectively reflect the stress response of HDPE specimen to temperature, type I specimen with small overall tensile strength was selected for the comparative tests. In the case of 3% elongation (all in elastic stage), the relationship between the tensile strength and the temperature of HDPE specimen and the fitting curve are obtained, as shown in Figure 5. With a decrease in temperature, the tensile strength of HDPE specimen increases obviously, which is approximately linear (R^2^ = 0.9986).

**Figure 6:**
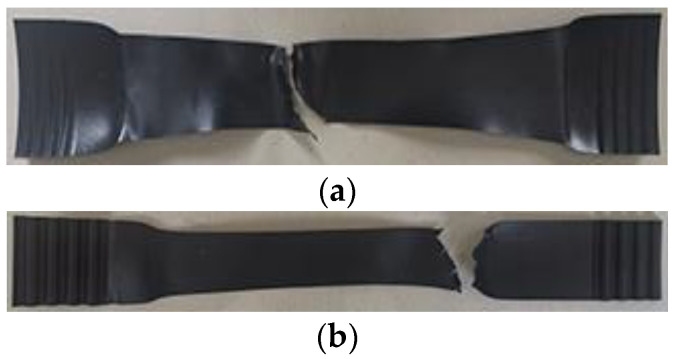
Fracture modes of the HDPE specimens. (**a**) Type I; (**b**) Type II.

The HDPE specimen fractures during a tension at −35 °C, as shown in Figure 6. The fracture mode of HDPE I and II specimens is similar and belongs to the ductile tearing failure. When a certain stress is reached, a small crack begins to appear on one side of the specimen. With an increase in displacement, the crack expands rapidly along the direction perpendicular to the axial force until the specimen is broken.

**Figure 7:**
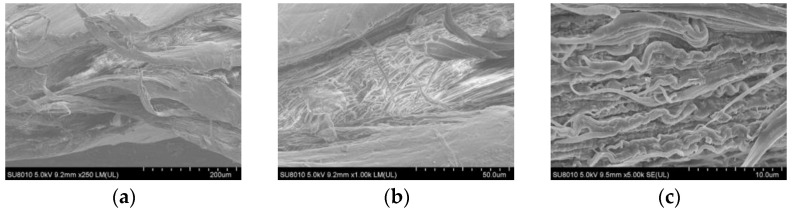
Microstructure of fracture surface of the HDPE specimens. (**a**) SEM image of 250 times; (**b**) SEM image of 1000 times; (**c**) SEM image of 5000 times.

By scanning electron microscopy (SEM), the fracture surface morphology of HDPE specimens after amplifications of 250, 1000, and 5000 times is shown in Figure 7. It was found that the fracture surface of HDPE specimens is rough, and the fiber bundles are complex. The compact and dense fiber bundles can be observed with a lens of 1000 times magnification, which can reach tens of microns. The fracture morphology magnified by a lens of 5000 times nearly presents a wave shape, and its characteristic is consistent with that of the viscoelastic failure during which obvious plastic deformation occurs.

### 3.2. Stress–Strain Relationship and Fracture Mode of PP Specimens

**Figure 8:**
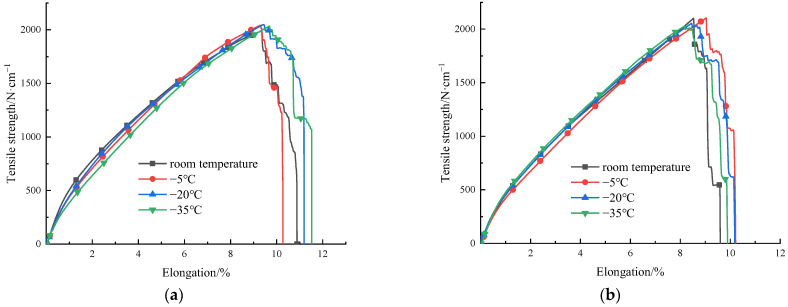
Tension curves of PP specimens I (**a**) and II (**b**) respectively at different temperatures.

**Figure 9:**
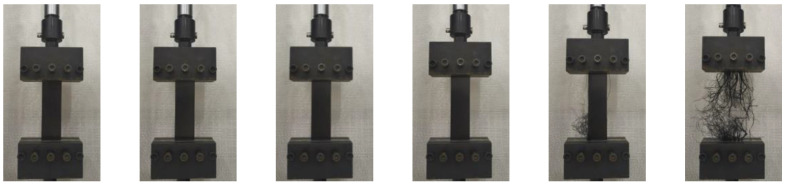
The tension process of a PP specimen.

The stress–strain relationship at different temperatures and the tension process of PP specimens are shown in Figure 8 and Figure 9, respectively. The trend of stress–strain relationships of type I and type II specimens is roughly the same. At the beginning of specimen loading, the stress increases rapidly with an increase in elongation. It can be seen that the failure of PP specimens conforms to the fracture mode of brittle materials. The yield and strengthening stages do not appear on the stress–strain relationships. When the tensile force reaches an allowable strength, the material fractures immediately. However, some of the fiber filaments are not broken after fracture, and a residual stress still exists. Therefore, it can be seen that the stress still has a slight upward–downward trend during the decline stage of stress–strain relationships. However, with increasing elongation, the specimen is finally broken.

When the temperature changes from high to low, the tensile strengths of type I specimens are 1989.1 N·cm^−1^, 2042.6 N·cm^−1^, 2047.45 N·cm^−1^, and 2034.95 N·cm^−1^; and those of the type II specimens are 2101.6 N·cm^−1^, 2104.4 N·cm^−1^, 2054.35 N·cm^−1^, and 2028.15 N·cm^−1^, respectively (Figure 8). It can be seen that the tensile strength of type I and type II specimens does not show obvious regularity with the change of temperature, indicating that the tensile strength of PP specimens is less sensitive to temperature. However, there is a slight difference in the stress–strain relationship of type I specimens in the elastic stage, which is due to the curling phenomenon of the short side of the PP specimens. An uneven stress at the clamp mouth of the jig is easy to produce, and the width of type I specimen is greater than that of the type II specimen. Therefore, the stress distribution at the jig of type I specimen is less uniform than that of type II, resulting in the concentration of tensile test results of type I specimen not being higher than that of the type II specimen. Based on the above analysis, it is concluded that the test data of type II specimen are more accurate.

**Figure 10:**
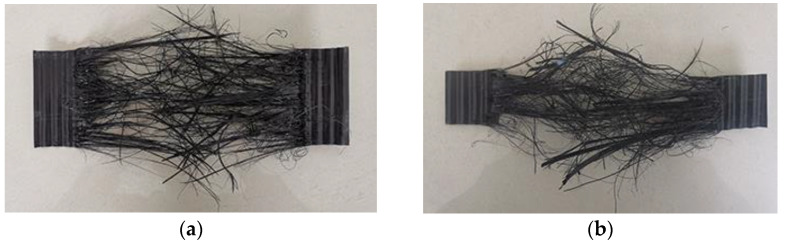
Fracture modes of the PP specimens. (**a**) Type I; (**b**) Type II.

The fracture mode of the PP specimens is shown in Figure 10. When the specimen reaches the tensile strength, crimped filaments are first stripped from the body along the length, accompanied by a crisp sound. Subsequently, the filaments on both sides gradually increase, and the sound gradually becomes disorderly. Finally, the original smooth surface suddenly explodes, and the specimen is completely broken. From the microscopic point of view, the brittle fracture of PP material originates from the long fibers. After a fracture of the long fibers, the fibers with poor orientation and short fibers continue to fracture, resulting in an instability of the material. At room temperature, inner micro pores in are the main reason for fracture of a PP specimen. Under the external force, these existing micro pores are expanded with micro cracks, resulting in the ultimate brittle cracking of the material. Under a combined action of low temperature and external force, the microfiber in the specimen is extracted from the matrix, which further leads to a brittle fracture of the material. From a macro point of view, PP specimens are subject to fibrous splitting failure.

**Figure 11:**
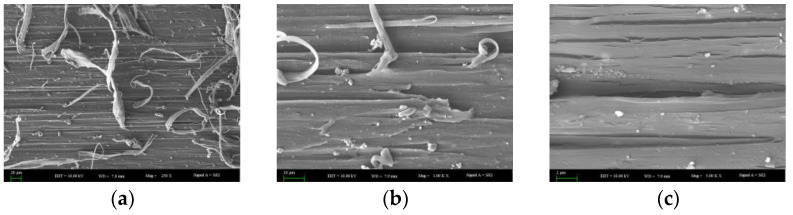
Microstructure of fracture surface of the PP specimens. (**a**) SEM image of 250 times; (**b**) SEM image of 1000 times; (**c**) SEM image of 5000 times.

By scanning electron microscopy (SEM), the fracture surface morphology of the PP specimens after amplifications of 250, 1000, and 5000 times is shown in Figure 11. Amplified by 250 and 1000 times with a lens, tearing microfiber bundles on the fracture surface of a PP specimen can be captured, but the whole arrangement is neat along the tensile direction. This finding conforms to the characteristics of brittle failure. According to the macroscopic and microscopic observations of the fracture surface, it was found that the length of the fiber broken on the fracture surface of the PP specimen is different, so the fracture surface of the PP specimen is an inclined plane.

### 3.3. Stress–Strain Relationship and Fracture Mode of PET Specimens

**Figure 12:**
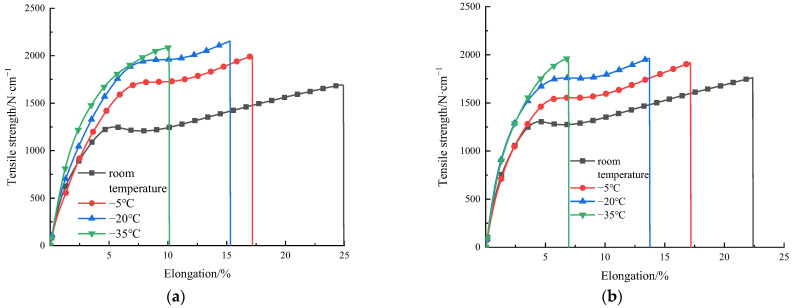
Tension curves of PET specimens I (**a**) and II (**b**) respectively at different temperatures.

**Figure 13:**
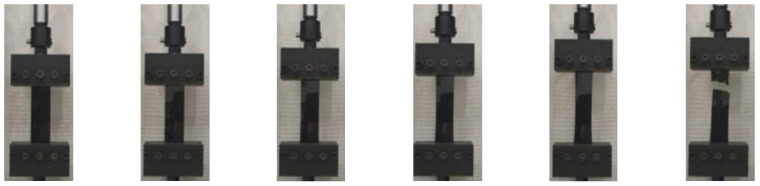
The tension process of a PET specimen.

The tension curves at different temperatures and the tension processes of PET specimens are shown in Figure 12 and Figure 13, respectively. The tension curve trend of type I and type II specimens is roughly the same. In the initial stage, with an increase in tensile force, the material is in the elastic stage because the elastic modulus of the material is almost unchanged at this stage, and the structure is stable. With an increase in elongation, the tension curve peaks at the yield point, and then enters the short strain softening stage immediately with the decrease in stress. Finally, it enters the strain hardening stage. At this stage, the material width is slightly contracted, but the necking phenomenon is not obvious. The specimen is finally broken. During the tension process at −35 °C, the trend of tension curves differs. The stress yield point does not appear on the tension curve, but the tensile strength increases with the displacement. When the stress reaches the allowable stress of the material, fracture occurs suddenly. The molecular chain of PET is composed of phenyl, ester, and aliphatic hydrocarbon groups, which have a good flexibility and certain rigidity. The material property is between toughness and brittleness. Under the tension at low temperature, the properties of PET material change, and the flexible chain effect decreases. The molecular motion energy is low, and the molecular chain is easier to break. At this point, the necking phenomenon no longer appears, and the curve does not have a strengthening stage, but the specimen is pulled out instantly.

**Figure 14:**
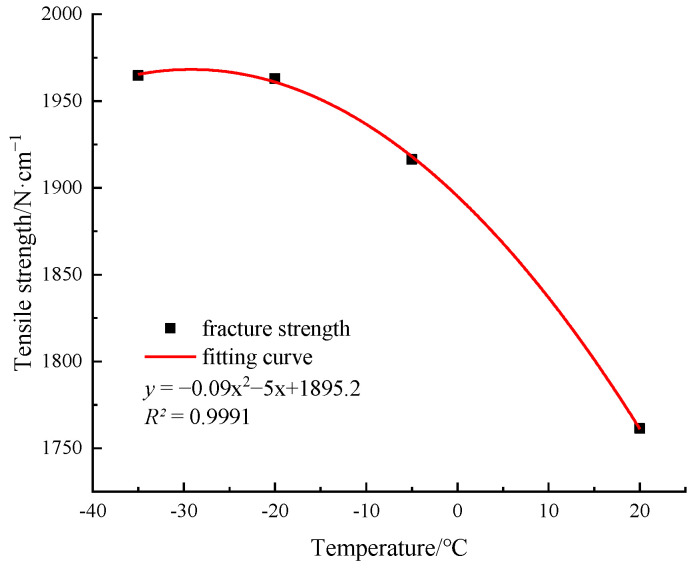
The tensile strength-temperature curve of the PET specimen.

As shown in Figure 12, the elongation decreases with a decrease in temperature. At the room temperature, −5 °C, and −20 °C, the tensile strength shows a stable upward trend, indicating that the tensile strength of PET materials is sensitive to the low temperature. The tensile strength of type I and type II PET specimens decreases from the room temperature to −20 °C, and the tensile strength is increased by 27.1% and 21.4%, respectively. However, when the temperature decreases from −20 °C to −35 °C, the tension curves of the two specimens shows the brittle fracture characteristics. The tensile strength of the type I specimen is decreased by 3.3%, and the tensile strength of the type II specimen is almost the same as that at −20 °C. To objectively reflect the stress response of PET material to temperature, the type II specimen with a small overall tensile strength is selected for the comparative tests. The relationship between the tensile strength and the temperature of the PET specimen and the fitting curve are shown in Figure 14. The tensile strength of PET specimen increases with a decrease in temperature. After reaching −20 °C, the fitting curve is nearly gentle, and can be described by a quadratic function (R^2^ = 0.9991).

**Figure 15:**
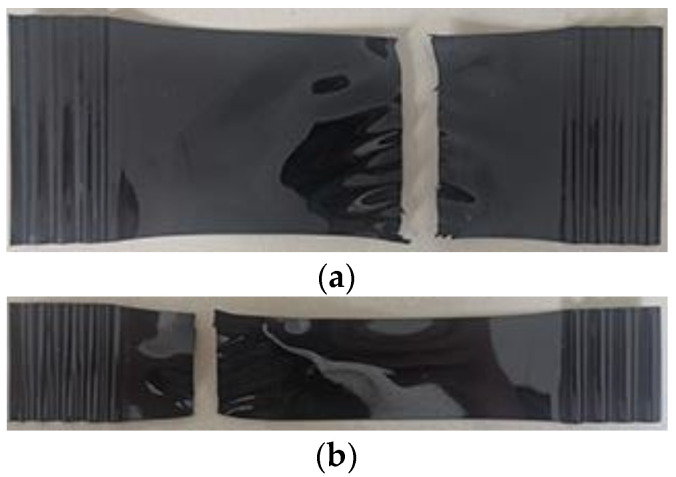
Fracture modes of the PET specimens. (**a**) Type I; (**b**) Type II.

The fracture mode of PET specimens is shown in Figure 15. The property of the PET specimen is between the toughness and brittle fracture, and the necking phenomenon during tension is not obvious. When the stress reaches a certain value, the material is suddenly broken. However, it can still be seen that the specimen fractures from one side, and there is a crack initiation zone. The fracture surface is relatively smooth and behaves in a straight line perpendicular to the axial force.

**Figure 16:**
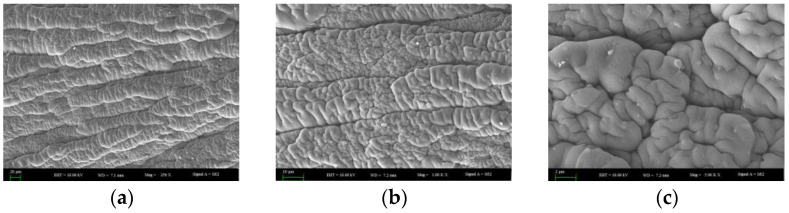
Microstructure of fracture surface of the PET specimens. (**a**) SEM image of 250 times; (**b**) SEM image of 1000 times; (**c**) SEM image of 5000 times.

By scanning electron microscopy (SEM), the fracture surface morphology of the PET specimens after amplifications of 250, 1000, and 5000 times is shown in Figure 16. The fracture surface of the PET specimen is rich in texture and layered stacking, but it is overall smooth without rough fibers. This is because the material extends to the crack propagation area immediately after cracks occur, resulting in a sudden fracture. Therefore, plastic deformation does not form on the fracture surface, and the failure of the PET specimen normally agrees with the basic morphological characteristics of a brittle fracture from the microscopic morphology of fracture surface.

### 3.4. Comparative Analysis of Test Results

Through an analysis of the stress–strain relationships of the three types of geocell specimens, it was found that their overall tensile properties are different. First of all, the production process of the three materials is different. The production process of the HDPE specimen is extrusion, and the specimen can experience a high elongation in the tension process. The main reason is that the HDPE specimen is not fully stretched in the production process, and the molecular chain orientation is low. When the material yields, the molecular chain begins to orientate, and the interaction between them increases. When the tensile stress increases to a certain extent, the molecular chain is broken, and the specimen is pulled off. This is also the reason why the HDPE specimen has a good ductility but a low strength. The PP and PET specimens are produced under tension, and the molecular chain is fully oriented along the tensile direction. This improves the tensile strength of the material. Secondly, the molecular chain configurations of the three materials are different. The HDPE has long chain aliphatic hydrocarbons, and the molecular chain is neat and compliant, whereas the intermolecular force is small. The PP has a regular structure and a high strength, but the flexibility of molecular chain is poor due to the existence of side methyl groups. The PET has a symmetrical benzene ring structure, and the phenyl is in a rigid group. The PET also has a flexible aliphatic hydrocarbon, so the macromolecular chain of PET is flexible and rigid.

The elongation and lateral deformation of three types of geocell specimens have been analyzed. Compared with the PP and PET specimens, the HDPE specimen produces the largest elongation at four test temperatures. However, there is an obvious necking phenomenon, and the lateral deformation is large. This leads to a premature failure of the material. The elongation of the PP specimen is less sensitive to temperature, and the range of elongation at failure is 8.17~9.04% at four test temperatures without lateral deformation. During the tension process at the room temperature, −5 °C, and −20 °C, the elongation of the PET specimen at failure was higher than that of the PP specimen. When the temperature is −35 °C, the elongation at failure is 6.94%, which is lower than the minimum elongation of PP specimen at failure (1.23%). The lateral necking deformation of the PET specimen is not obvious.

Through an analysis of the micro-morphology of fracture surfaces of the three geocell specimens, the fiber lengths of fracture surfaces of the HDPE specimens are found to be different. The whole micro-morphology is rough, which conforms to the feature of ductile tearing failure. The microfiber bundles on the fracture surface of the PP specimen are neatly arranged along the tensile direction, and some fibers are crimped after a fracture. The fracture surface is an inclined plane, and the failure of the PP specimen belongs to a brittle failure. The fracture surface of the PET material is relatively smooth, with no fracture fiber, and conforms to the features of a brittle failure.

**Figure 17:**
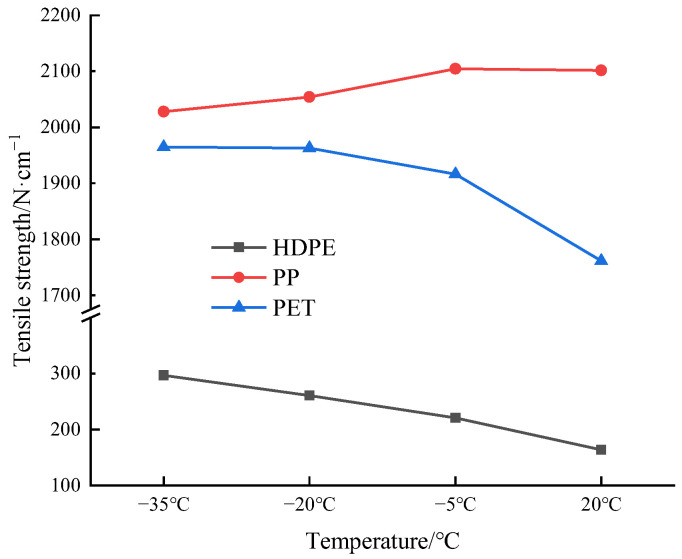
Tensile strength-temperature curves of the three geocell specimens.

According to the tensile test results of the three geocell specimens, a relationship between the temperature and the tensile strength has been obtained, as shown in Figure 17. The tensile strength of HDPE specimen is the lowest among those of the three specimens, and the tensile strength of PET is slightly lower than that of PP specimen. From the room temperature to −35 °C, the tensile strengths of HDPE and PET specimens increase, but the effect of temperature on the tensile strength of PP specimens is not obvious.

The tensile strength of the PP specimens is high and nearly unaffected by the low temperature, but the lateral flexural strength of the PP specimens is extremely low, and the material is brittle as a whole. The toughness of the PET specimens is higher than that of the PP specimens, and its strength is higher than that of the HDPE specimens. With a decrease in temperature, the tensile strength increases, and the elongation at failure is higher than that of the PP specimen as a whole. Only when the temperature is equal to −35 °C, the elongation of PET specimens at failure is 1.23% lower than that of the PP specimen. In summary, the PET specimens outperform the HDPE and PET specimens as a whole. In the cold environment, PET specimens have more advantages in tensile properties.

## 4. Conclusions

Through groups of uniaxial tensile tests on geocell strips, the tensile properties of the HDPE, PP, and PET geocell strips at low temperature were compared and analyzed. Firstly, the strength and deformation characteristics of three kinds of geocell strips at low temperature were analyzed. Secondly, scanning electron microscopy was used to study the fracture mode of each geocell strip at the microscopic scale. Finally, according to the sensitivity of three kinds of materials to temperature, the geocell strip with better performance in all aspects under low temperature environment was given. It is convenient to further explore the reinforcement mechanism of geocell in cold area engineering. The conclusions are as follows:

- The elongation of the HDPE geocell strip is the largest, but the necking phenomenon occurs during the tension process. The PET and PP geocell strips have no obvious deformation during tension.
- The HDPE geocell has a good ductility, and the plastic deformation on fracture surface is obvious, which belongs to a ductile failure. At the room temperature, −5 °C, and −20 °C, the PET geocell strip has yield and strengthening stages, and the fracture surface is relatively smooth, which presents the feature of a hard ductile failure. However, at a temperature of −35 °C, the failure pattern of PET geocell strip turns to the brittle failure. There is no plastic deformation on the fracture surface of the PP geocell strip under various temperature conditions, which conforms to the feature of brittle failure.
- When the temperature decreases from the room temperature to −35 °C, the tensile strengths of the HDPE and PET strips are increased by 53% and 21.4%, respectively, while the tensile strength of PP geocell strips has no obvious regularity. The temperature sensitivity of the three geocell strips from high to low is as follows: HDPE, PET, and PP.
- The elongation of PET strip at failure is higher than that of the PP strip at the room temperature, −5 °C, and −20 °C, and the elongation of the PET strip at failure is slightly lower than that of the PP specimen at −35 °C. In cold regions, the overall tensile properties of the PET geocell specimens are more favorable than those of HDPE and PP geocell specimens.

## Figures and Tables

**Table 1:** Parameters of geocell strips.

Material	Specimen Width/mm	Specimen Thickness/mm	Length of Gauge/mm
HDPE	100	1.1	20
PP	100	0.5	20
PET	100	0.5	20

Note: HDPE refers to the high-density polyethylene, PP denotes the polypropylene, and PET represents the polyethylene terephthalate.

## Data Availability

No new data were created or analyzed in this study; data sharing is not applicable to this article.
